# Human Very Small Embryonic-Like Stem Cells Are Present in Normal Peripheral Blood of Young, Middle-Aged, and Aged Subjects

**DOI:** 10.1155/2016/7651645

**Published:** 2015-11-08

**Authors:** Hanna Sovalat, Maurice Scrofani, Antoinette Eidenschenk, Philippe Hénon

**Affiliations:** Institut de Recherche en Hématologie et Transplantation, Hôpital du Hasenrain, 87 avenue d'Altkirch, 68100 Mulhouse, France

## Abstract

The purpose of our study was to determine whether the number of human very small embryonic-like stem cells (huVSELs) would vary depending on the age of humans. HuVSELs frequency was evaluated into the steady-state (SS) peripheral blood (PB) of healthy volunteers using flow cytometry analysis. Their numbers were compared with volunteers' age. Blood samples were withdrawn from 28 volunteers (age ranging from 20 to 70 years), who were distributed among three groups of age: “young” (mean age, 27.8 years), “middle” (mean age, 49 years), and “older” (mean age, 64.2 years). Comparing the three groups, we did not observe any statistically significant difference in huVSELs numbers between them. The difference in mRNA expression for PSC markers as SSEA-4, Oct-4, Nanog, and Sox2 between the three groups of age was not statistically significant. A similar frequency of huVSELs into the SS-PB of young, middle-aged, and aged subjects may indicate that the VSELs pool persists all along the life as a reserve for tissue repair in case of minor injury and that there is a continuous efflux of these cells from the BM into the PB.

## 1. Introduction

Under steady-state conditions, small amounts of hematopoietic stem cells (HSCs) constantly leave the bone marrow (BM), penetrate the tissues, and return to the BM or peripheral niches via the blood or the lymphatic system [1–3]. Thus, the peripheral blood (PB) may be envisioned as a highway by which HSC can relocate between distant stem cell niches to keep the pool of BM stem cells in balance. In adult organisms, circulating stem/progenitor cells show a circadian rhythm with a peak occurring early in the morning and a nadir at night. Their number increases modestly during minor hematopoietic stresses, such as infection or strenuous exercise, [4–6]. In addition to HSCs, some rare other stem/progenitor cells such as mesenchymal stem cells (MSCs) [7], fibrocytes [8, 9], skeletal progenitors [10, 11], and endothelial progenitor cells (EPCs) [12–14] may also circulate into the PB after tissue/organ injury. Recently, a population of very small embryonic-like stem cells (VSELs) was identified in murine adult bone marrow [15]. Murine VSELs (muVSELs) are small, nonhematopoietic cells with high nuclear/cytoplasm ratio and unorganized euchromatin and express markers of pluripotent embryonic and primordial germ cells. MuVSELs have been identified in most tissues [16]. These cells circulate in a very few number into the PB under steady-state (SS) conditions and in much larger numbers after administration of granulocyte-colony stimulating factor (G-CSF) [17]. It was demonstrated that, either under steady-state conditions or after response to G-CSF, circulating VSELs numbers were lower in older animals than in younger ones [18, 19]. Human VSELs (huVSELs) have been firstly identified in umbilical cord blood as CXCR4^+^, CD34^+^, CD133^+^, Lin^−^, and CD45^−^ cells enriched for Oct-4 and SSEA-4 [20]. We have recently shown that a similar population of huVSELs was present in both adult BM and PB and could be harvested by leukapheresis after G-CSF administration [21].

However, whether the number of VSELs would also vary in humans depending on the age has to be determined. In the present study, we have both assessed the presence of huVSELs into the steady-state PB of healthy volunteers and compared their numbers in function of age.

## 2. Material and Methods

### 2.1. Human Healthy Volunteers

28 healthy volunteers (12 females and 16 males; mean age, 41.9 ± 15.4 years; range 20–70 years), not taking any medication, were enrolled in this study after informed consent.

20 mL of SS-PB was withdrawn by venous puncture from each subject early in the morning to avoid the impact of any physical effort on PB cell counts, collected on EDTA, and immediately processed. The absolute number of white blood cells (WBC) was determined at the same time using a Coulter A^c^T diff cell counter (Beckman Coulter, Roissy, France).

### 2.2. Flow Cytometry (FCM) Analysis

Staining and FCM analysis were performed as previously described [21]. Briefly, samples of whole PB were lysed in hypotonic ammonium chloride buffer (IOTest lysing solution, Beckman Coulter, Roissy, France) to remove red blood cells. Total nucleated cells (TNC) were stained with a mixture of lineages (Lin) associating monoclonal antibodies (MoAbs) conjugated with fluorescein isothiocyanate (FITC). At the same time, phycoerythrin (PE) conjugated-CD45 MoAb clone J33 (Beckman Coulter, Roissy, France) and a combination of allophycocyanin (APC) conjugated MoAbs, CD133 clone AC133 (Miltenyi Biotec, Paris, France), CD34 clone 8G12, or CD184 (CXCR4) clone 12G5 (BD, Le Pont de Claix, France), were added for 30 minutes on ice. Cells were then washed and fixed with 4% formaldehyde (FA) for 20 minutes. Finally, 7-aminoactinomycin D (7-AAD; BD, Le Pont de Claix, France) was added to stain nucleated cells.

FCM analyses were performed using a FACSVantage DIVA fluorescence-activated cell sorting device (BD Biosciences, Erembodegem, Belgium). At least 10^6^ small events ranging from 2 to 10 *μ*m were included in the gate after comparison with five different size beads calibrated at standard diameters of 1, 2, 4, 6, and 10 *μ*m (Flow Cytometry Size Calibration, Invitrogen/ThermoFischer Scientific, Illkirch, France). CXCR4^+^ Lin^−^ CD45^−^, CD34^+^ Lin^−^ CD45^−^, or CD133^+^ Lin^−^ CD45^−^ cell subset amounts were counted among the nucleated 7-AAD^+^ cells.

Cell subpopulations absolute numbers were calculated in 1 mL of PB.

### 2.3. Reverse Transcription-Quantitative-Polymerase Chain Reaction (RT-qPCR) Analysis

RNA extraction from total PB-NC and analysis of* Oct-4*,* Nanog*, and* Sox2* mRNA were carried out as previously described [21]. Primer sequences of* Oct-4*,* Nanog*,* Sox2*, and *β*_2_ are summarized in Table 1. RNA isolated from H9 and HUES 3 hESC lines [22, 23], kindly provided by the “Plateforme Cellules Souches Embryonnaires Humaines” (Inserm U602 Villejuif, France), was used as reference sample for each PCR reaction.

### 2.4. Immunofluorescence Staining

The expression of pluripotency antigens was determined for each healthy volunteer. NC were stained for 2 hours with antibodies against SSEA-4 (clone MC-813-70, mouse monoclonal IgG), Tumor Rejection Antigen (TRA-1-81, clone TRA-1-81, mouse monoclonal IgM), OCT-4A/4B (clone 9E3.2, mouse monoclonal IgG), and NANOG (goat polyclonal) (Millipore, Molsheim, France), as previously described in detail [21]. Appropriate secondary FITC or tetramethylrhodamine-5-isothiocyanate (TRITC) goat anti-mouse IgG or IgM and FITC-goat anti-rabbit (Beckman Coulter, Marseille, France) were added for 1 h. The nuclei were labelled with 4′,6-Diamidino-2-phenylindole (DAPI) complemented with Vectashield (Vector Laboratories, Abcys, Paris, France). Cells stained with secondary antibodies only were used as negative controls. Slides with H9 and/or HUES3 hESC lines were stained similarly and used as positive controls. Fluorescence images were recorded with the AxioVision 4.7 “Full support” system attached to a fluorescent microscope Axiostar Plus Zeiss and captured by AxioCam ICC 1 R3 Cameras (Lordil, Villers-les-Nancy, France).

### 2.5. Statistical Analysis

Data were expressed as mean ± standard deviation (SD). To verify the difference in expression of each marker between the different age groups, normality data were tested by the Shapiro test. Once normality is verified, one-way ANOVA was chosen to carry out this comparison; on the contrary, the Kruskal-Wallis test was chosen when normality was not verified. *t*-test pairwise analysis was performed when previous tests were significant.

## 3. Results

### 3.1. VSELs Numbers Do Not Decrease with Aging

**Figure 1:**
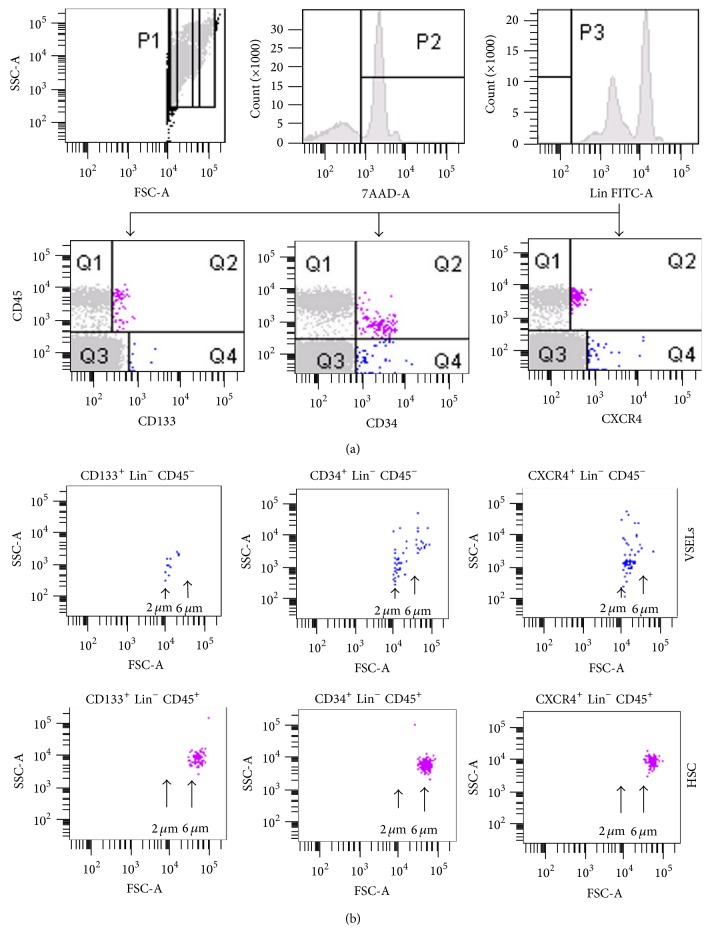
FCM analysis of PB-derived VSELs. Erythrocytes were removed by hypotonic lysis and PB NCs were stained with fluorescein isothiocyanate-lineage markers (Lin), phycoerythrin-CD45 and allophycocyanin-CD133, CD34, or CXCR4 mAbs. (a) Left dot plot: 10^6^ events ranging from 2 to 10 *µ*m were included in the P2 gate. Right histogram: Lin^−^ cells were included in the P1 analysis gate. Middle histogram: P1 gated cells stained with 7-aminoactinomycin D (7-AAD) were included in the P2 gate. Right histogram: Lin^−^ cells were included in the P3 gate and were analyzed for CD45 coexpression with CD133, CD34, or CXCR4 antigens. (b) Dot plots showing the size of analyzed CD133^+^, CD34^+^, or CXCR4^+^, Lin^−^, CD45^−^ VSELs (upper row) and CD133^+^, CD34^+^, or CXCR4^+^, Lin^−^, CD45^+^ HSC (lower row).

Enumeration of circulating VSELs requires FACS-unique gating strategies to focus on very rare and small events (Figures 1(a) and 1(b)).

We applied here the identification strategy which was published in detail by Zuba-Surma and Ratajczak [24]. Three different subpopulations of huVSELs (diameter ranging from 3 to 6 *μ*m) have thus been detected into SS-PB but in very low numbers. Considering altogether the healthy adult volunteers enrolled in this study, 290 ± 150 CD133^+^ Lin^−^ CD45^−^ cells, 80 ± 60 CD34^+^ Lin^−^ CD45^−^ cells, and 300 ± 260 CXCR4^+^ Lin^−^ CD45^−^ cells were counted on average per mL of total blood.

**Figure 2:**
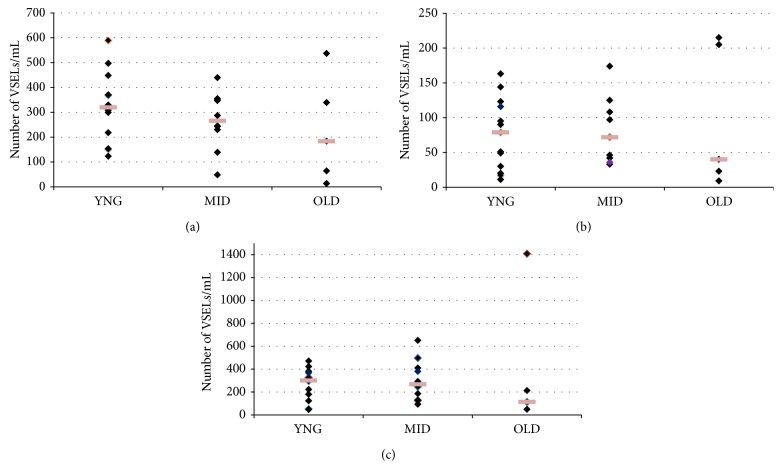
Age-dependent frequency of Lin^−^/CD45^−^ cell subsets expressing (a) CD133, (b) CD34, and (c) CXCR4 into the PB. Three groups of healthy human volunteers were designed according to their age: “young” (20–39 years; *n* = 13), “middle” (40–59 years; *n* = 10), and “older” (60–70 years; *n* = 5). Frequency of Lin^−^ CD45^−^ cell subsets was calculated per mL of PB. Each square represents the number of VSELs/mL per volunteer in each group. The difference between the three groups of volunteers is not statistically significant (*P* > 0.05; one-way analysis of variance (ANOVA)).

When volunteers were distributed among three groups of age, “young” (mean age, 27.8 years, range from 20 to 39 years; *n* = 13), “middle,” (mean age, 49 years, range from 40 to 59 years; *n* = 10), and “older” (mean age, 64.2 years, range from 60 to 70 years; *n* = 5) groups, not any statistically significant difference in huVSELs frequency (expressed as number of cells/mL) in PB was observed between these three groups (*P* > 0.05; one-way analysis of variance (ANOVA)) (Figure 2).

However, in the older group, all VSELs subset numbers were lower in 3 of the subjects compared to the other 2, but this decline was not statistically significant. It may be due to the low number of “old” patients (*n* = 5) compared to the number of “young” and “middle-aged” groups. It is clearly important to increase the number of patients in the “old” group to have a clear data. This low number of aged healthy volunteers is due to our difficulty to find people with advanced age taking any drug. In fact, in our future investigations, by increasing our collaborations with other laboratories we hope to resolve this problem.

### 3.2. A Population of Small CD45^−^ Cells Expressing Several Pluripotent Stem Cell Markers Is Present in SS-PB of Young, Middle, and Older Subjects

**Figure 3:**
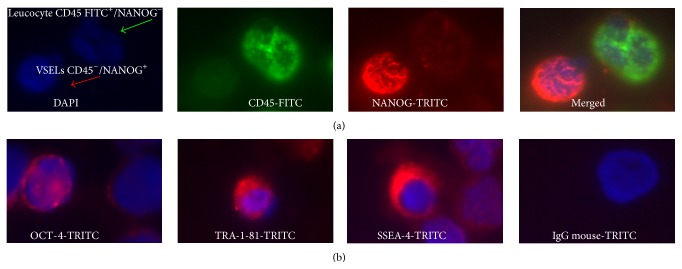
Immunofluorescence analysis of PB-derived VSELs. A typical triple-staining with 4′,6-diamidino-2-phenylindole (DAPI) (blue: nuclei), fluorescein isothiocyanate- (FITC-) CD45 (green fluorescence), and tetramethylrhodamine-5-isothiocyanate- (TRITC-) SSEA-4^−^, TRA-1-81, OCT-4, or NANOG (red) shows (a) VSELs: small CD45^−^ cells NANOG^+^ and leukocytes: greater CD45^+^ NANOG^−^ cells and (b) VSELs: CD45^−^ cells which express OCT-4 in nuclei or TRA-1-81 and SSEA-4 on the surface.

Immunofluorescence staining showed that CD45^−^ cell subsets express both specific pluripotent stem cell (PSC) markers such as SSEA-4 and TRA-1-81 on their surface and OCT-4 and NANOG transcription factors at the protein level (Figure 3).

**Figure 4:**
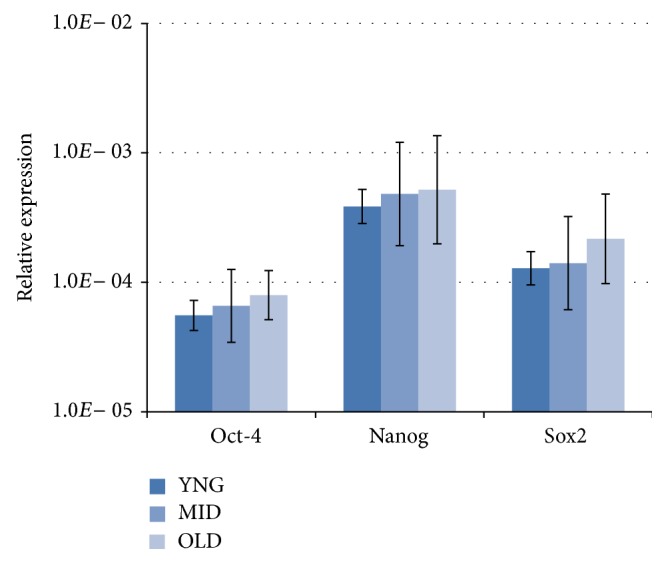
Relative expressions of PSC markers, Oct-4, Nanog, and Sox2, were measured by RT-qPCR and compared using equal amounts of mRNA isolated from CNT PB of young, middle, and older healthy volunteers. The relative expression of each PSC marker is calculated according to a positive control PCR (RNA isolated from H9 and HUES 3 hESC lines). Data represent the mean ± standard deviation for each age group. The difference in mRNA expression of these markers between the three groups of age was not statistically significant (*P* > 0.05, Kruskal-Wallis test).

Expression of PSC markers was confirmed by RT-qPCR. The differences in mRNA expression for those markers between the three groups of age were not statistically significant (*P* > 0.05, Kruskal-Wallis test) (Figure 4).

## 4. Discussion

The PB could be envisioned as a highway by which stem cells are trafficking in the body to keep in balance a pool of stem cells located in different niches in peripheral tissues. In this context, the BM has been proposed to be a main reservoir for these circulating cells [25]. In addition to HSCs, several other types of stem/progenitor cells have been described in the adult BM, such as mesenchymal stem cells (MSCs) [26], marrow-isolated adult multilineage inducible (MIAMI) cells [27], and multipotent adult stem cells (MASCs) [28]. A population of VSELs was first identified in the murine BM by the group of Ratajczak [15]. These cells express several markers characteristics for embryonic stem cells (ESCs), such as Oct-4, Nanog, and SSEA-1. MuVSELs circulate at very low numbers (~150–300/mL) into the murine SS-PB. Wojakowski et al. have also recently shown that very small numbers of VSELs (80–1300 cells/mL) could be detected under steady-state condition into the SS-PB of healthy humans, aged from 30 to 50 years, thus reflecting a continuous efflux of these cells from the BM [29]. Our present study, performed in a population of healthy adults with a larger age range (from 20 to 70 years), confirms that huVSELs actually circulate at very low levels (80–300/mL) into the SS-PB, in young and in older subjects. Furthermore, they can be physiologically mobilized in order to participate in tissue/organ repair. Indeed, in several pathological situations, both in mice and in humans (e.g., heart infarct, stroke, skin burn injury, Crohn's disease, etc.), VSELs are released into the PB and their circulating numbers thus significantly increase [30–34]. However, when VSELs are released from the BM as a physiological response to tissue/organ injuries, even if they are able to home to the damaged areas, it is likely that they can only participate in the regeneration of minor tissue injuries and not of the largest ones, because of their too small amounts. To improve their potential regenerative impact, murine and huVSELs could then be mobilized by G-CSF administration [17, 21]. We have thus observed that circulating huVSELs amounts increase up to 2–4-fold after 4 days of G-CSF administration [35].

In case of myocardial infarct, it was also noticed that the intensity of the pic of VSELs spontaneous mobilization correlates with the extend of the ischemic [36, 37] or stroke lesion [32]. The knowledge of time occurrence of the mobilization pic might thus allow the determination of a therapeutic “window” useful for clinical application. On the other hand, the spontaneous (equal to physiological) mobilization rate could reflect the overall “regenerative potential” of an adult organism. Reduced spontaneous stem cell mobilization has been associated with poor prognosis in patients with myocardial infarction [37]. Thus, we suggest that the mean steady-state level of circulating huVSELs determined from a large enough cohort of healthy subjects might serve as a reference value which could be used as a baseline from which the physiopathological VSEL mobilization rate following an organ injury (e.g., heart infarct or stroke) would be correlated with its severity and its prognosis.

We also focused our study on the potential effect of aging on the amounts of huVSELs circulating in PB under steady-state conditions. The effects of aging on the HSC compartment have been extensively studied in mice. In the murine HSC system, several studies have reported an increase in the absolute number of phenotypically defined HSCs, although elderly animals have a reduced repopulation potential when compared to younger organisms [38–42]. Additionally, other investigators interestingly showed in mice that the frequency of the HSC subpopulation phenotypically defined as lineage negative Sca-1^+^, c-kit^+^, Thy-1^+^, CD135^−^ side population (SP) steadily increased in the BM of the femurs and tibias with age. But, although long-term repopulation assays indicated that SP cells are still present and capable of self-renewal and differentiation in older mice, they have shown a lower homing efficiency than those from younger mice [43].

A little study on age-related changes in HSC number and function has been reported in humans. Although indirectly demonstrated through clinical data, there is evidence that human HSC function declines with age. For example, hematopoietic engraftment following HSCs transplantation is often faster and better sustained when the donor (in the autologous as well in the allogeneic setting) is young. Also, elderly people frequently and specifically develop hematological disorders due to acquired HSCs abnormalities, as, for example, myelodysplastic syndromes (MDS). Marley et al. have also found clear* in vitro* correlations between the relative numbers and function of clonogenic myeloid progenitors (CFU-GM) and age of the donor:* in vitro* colony formation study has indeed revealed that, although the absolute number of colony forming cells increases with age, their individual self-renewal capacity decreases [44].

Regarding VSELs, it was recently demonstrated in steady-state mice that, unlike HSC and SP, the number of muVSELs in the BM and the PB gradually decreases with age, in parallel with their ability to form VSEL-derived spheres (VSEL-DS) containing primitive stem cells, which may explain why the regenerative processes are less efficient in advanced age [18, 19]. Moreover, the number of VSELs is much higher in the BM of long-lived (C57BL/6) as compared to short-lived (DBA/2) mice [15], which allows supposing a positive correlation between high VSEL numbers and a greater length of life.

The effects of aging on the VSELs compartment had never been evaluated in humans until our present study. In response to G-CSF administration, we did not observe age-dependent huVSELs release into leukapheresis products from adult cancer patients aged from 34 to 71 years [35]. Here, we interestingly show for the first time that the numbers of circulating huVSELs in healthy subjects aged from 20 to 70 years were not statistically different whichever the age bracket is and thus would not seem to decline significantly with age. However, as we observed that the number of the all VSELs subpopulations was lower, while not significantly, in 3 subjects in the older group, this would nevertheless suggest that the frequency of CD133^+^, CD34^+^, or CXCR4^+^ VSELs subpopulations could progressively run out with aging. A study including more number of older subjects is necessary in future to support this hypothesis.

On the other side, it is supposed that, as we age, stem cells likely progressively lose at least a part of their self-renewal and/or differentiation capabilities, which would lead to reduce their cell tissue regeneration potential and consequently contribute to the global somatic senescence. Thus, it would be crucial to demonstrate whether the proliferation potential of huVSELs also depends on age in humans. However, while murine VSELs proliferation and differentiation has been well demonstrated* in vitro*, we and others had failed to demonstrate such proliferative capacities of huVSEls by* in vitro* studies up to now. The major difficulty is that these cells stay in dormant state and nobody had already identified the combination of factors necessary to trigger the expansion/differentiation processes in purified huVSELs* ex vivo* cultures. Hopefully, the Taichman's group has very recently shown that huVSELs are able to generate multiple tissues within an osseous wound in immune-deficient mouse when cocultured with the C2C12 cell line [45], thus demonstrating for the very first time* in vitro* that huVSELs have actually the capacity to self-renew.

## 5. Conclusions

In this study, we demonstrate that huVSELs circulate at a similar frequency into the SS-PB of young, middle-aged, and aged subjects. This may indicate that the pool of VSELs* persists along the life* as a reserve for tissue repair in case of minor injury, and that there is a continuous efflux of these cells from the BM into the PB. We hypothesize that in case of major health complications (e.g., heart infarct, stroke) circulating VSELs could be isolated even from elderly patients,* ex vivo* expanded, and reinjected back into the same recipients for therapeutic purposes. Of course, such a strategy should be first tested in animal models. First and foremost, the actual VSELs' ability to self-renew and to differentiate has to be determined before they could be used for cell-based clinical therapies. In the recent study, the Taichman's group now opens the way for future huVSEL-based regenerative therapies at least for osseous, neural, and connective tissue disorders.

## Figures and Tables

**Table 1:** Sequences of the forward and reverse primers employed for qPCR.

	Forward	Reverse
Oct-4	GAT GTG GTC CGA GTG TGG TTC T	TGT GCA TAG TCG CTG CTT GAT
Nanog	GCA GAA GGC CTC AGC ACC TA	AGG TTC CCA GTC GGG TTC A
Sox2	TAC AGC ATG TCC TAC TCG CAG	GAG GAA GAG GTA ACC ACA GGG
Beta-2-microglobuline	AAT GCG GCA TCT TCA AAC CT	TGA CTT TGT CAC AGC CCA AGA TA
